# Yiqi Huoxue Recipe inhibits cardiomyocyte apoptosis caused by heart failure through Keap1/Nrf2/HIF-1α signaling pathway

**DOI:** 10.1080/21655979.2021.1900634

**Published:** 2021-03-19

**Authors:** Ling Hu, Yanan Xu, Qian Wang, Meijie Liu, Linfeng Meng, Dongyan Yan, Huagang Hu, Minjia Xiao, Zhenzhen Yin, Ying Li, Xiaoping Kang

**Affiliations:** a Internal medicine, Beijing Xiaotangshan Hospital, Beijing, China; b Department of Cardiopulmonary Rehabilitation, Beijing Xiaotangshan Hospital, Beijing, China; c Rehabilitation Department, Beijing Xiaotangshan Hospital, Beijing, China; d Animal Laboratory, Medical Experimental Center, Chinese Academy of Chinese Medical Sciences, Beijing; e Research Laboratory, Beijing Xiaotangshan Hospital, Beijing, China

**Keywords:** Yiqi Huoxue Recipe (YHR), heart failure, cardiomyocyte apoptosis, keap1, nrf2, hif-1α

## Abstract

Yiqi Huoxue Recipe (YHR) is commonly used in China to treat diseases such as heart failure (HF). It has been reported that YHR can treat HF and has a certain protective effect on myocardial cell damage. The purpose of this study is to determine the cardioprotective effects of YHR on HF-induced apoptosis and to clarify its mechanism of action. Oxygen glucose deprivation/recovery (OGD/R) induces H9C2 cell apoptosis model. Ligation of the left anterior descending artery (LAD) coronary artery can induce an animal model of HF. We found that YHR protected H9C2 cells from OGD/R-induced apoptosis, reduced the level of reactive oxygen species (ROS) in H9C2 cells, and increased the mitochondrial membrane potential in H9C2 cells. The results of in vivo animal experiments showed that in the HF model, YHR could reduce infarct area of heart tissue and cardiomyocyte apoptosis rate. YHR regulated the expression of key apoptotic molecules, including increasing the ratio of Bcl-2 and Bax, and reducing the expression of Kelch-like ECH-associated protein 1 (Keap1) and caspase-3. Interestingly, YHR also regulates the expression of NF-E2-related factor 2 (Nrf2) in the nucleus. In summary, YHR may provide cardioprotective effects in heart failure through inhibiting the Keap1/Nrf2/HIF-1α apoptosis pathway.

## Graphical Abstract

**Figure:**
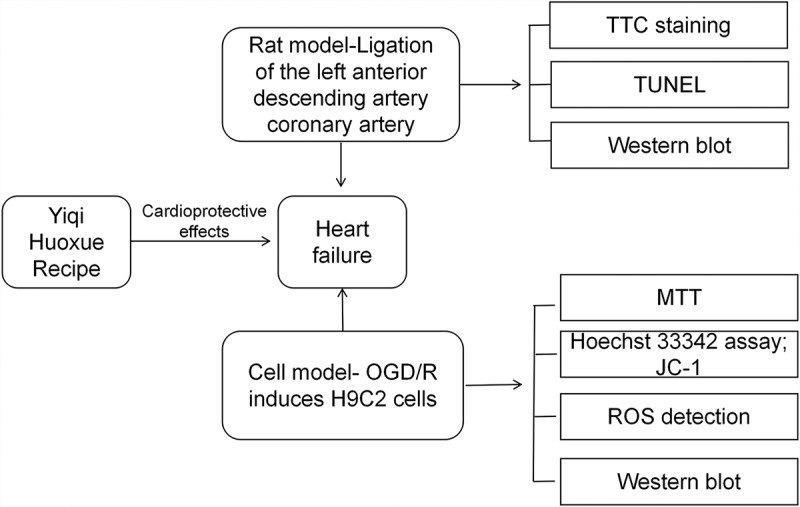

## Introduction

Chronic heart failure (HF) is a process of occurrence and development closely related to various pathophysiological processes and involving multiple mechanisms [1,2]. Its prevention and treatment are known as one of the common thorny problems in the field of medical treatment. In China, there are more than 4 million HF patients. Chronic heart failure develops to the later stage, and water-liquid metabolism disorder has become one of the most common clinical symptoms, which can lead to continuous impairment of heart function. Research on the mechanism of HF has been ongoing, and it has been proven that apoptosis can promote the progression of HF [3]. The pathophysiological mechanisms of heart failure are complex, such as activation of the renin-angiotensin-aldosterone system (RAAS), excessive activation of sympathetic nerves and inflammatory factors, and cardiomyocyte apoptosis. Studies have shown that the loss of cardiomyocytes caused by cardiomyocyte apoptosis is a key factor in the aggravation of heart failure, and inhibition of ventricular remodeling caused by cardiomyocyte apoptosis can improve the prognosis of heart failure and provide an effective and brand-new way for the prevention and treatment of heart failure [4,5]. Studies have reported that the apoptotic cells around the infarcted tissue caused by chronic heart failure play an important role in the process of heart remodeling. During the onset of HF, the apoptotic signaling pathway mediated by Keap1-Caspase-3 has important functions [6].

Kelch-like ECH-associated protein 1 (Keap1) is an NF-E2-related factor 2 (Nrf2) cytosolic inhibitory protein of the Kelch family. It mainly exists in the cytoplasm and is normally anchored on the cytoskeleton of cytoplasmic actin. Keap1 can regulate cell apoptosis and play a role in many diseases [7]. Keap1 can up-regulate Bax and inhibit the effect of Bcl-2 in preventing apoptosis [8]. The target gene regulated by Nrf2 plays a key role in cardiac function damage and cardiac remodeling. Nrf2 deletion or expression inhibition can increase the susceptibility of cardiomyocytes and cardiac fibroblasts to oxidative stress and cytotoxicity of oxygen-free radicals [9]. In addition, when the body is attacked by reactive oxygen species (ROS), it can inhibit the synthesis of protective protein through the regulation of the Keap1-Nrf2-HIF-1α pathway, thereby causing damage to the body’s cells [10]. Therefore, reducing the expression of Keap1 protein by interference means to inhibit the apoptosis of cardiomyocytes in HF will provide a new reference plan for the prevention and treatment of HF.

The components of traditional Chinese medicine have a protective effect on the cardiovascular system, and have the characteristics of small side effects, suitable for long-term use, and have a good effect on the treatment of cardiovascular diseases. Yiqi Huoxue Recipe (YHR) is one of the traditional Chinese medicine formulas, including aloe-emodin, rhein, emodin, chrysophanol, emodin methyl ether, which can enhance myocardial contractility, increase coronary flow, improve heart function, and reduce myocardial ischemia-reperfusion injury [11,12]. YHR can protect the myocardium by improving the body’s sensitivity to the oxidative stress system and enhancing the protective proteins glutathione peroxidase (GPX) and catalase (CAT) produced after the activation of the oxidative stress system to reduce the damage of myocardial cells [13]. YHR can mediate the prevention of HF by reducing oxidative stress and inhibiting apoptosis [11]. However, the anti-apoptotic effect of YHR has not yet been elucidated. In this study, we conducted in vivo and in vitro experiments to study the potential mechanism of YHR inhibiting cardiomyocyte apoptosis in HF.

## Materials and methods

### Cell culture

H9C2 cells were purchased from the Shanghai Institute of Cell Biochemistry, Chinese Academy of Sciences (China), and used 100 IU/mL penicillin and 100 μg/mL streptomycin solution containing 10% fetal bovine serum (FBS, purchased from GIBCO) in DMEM medium (HyClone company) training.

## Materials

Tiron was from Sigma Aldrich (USA). MTT, Dimethyl sulfoxide (DMSO), DCFH-DA, 2,3,5-Triphenyltetrazolium chloride (TTC) staining were purchased from Sigma (USA). The kits contain TdT10x, fluorescein-labeled dUTPlx, and fluorescein antibody-labeled HRP were obtained from Roche (Switzerland). JC-1 kit and Hoechst33,342PI cell apoptosis staining kit were purchased from Beijing Dingguo Biotechnology Co., Ltd. (China). Antibodies for Nrf2, caspase-3, KEAP1, Bcl-2, and Bax were all from Abcam (UK). The GAPDH antibody and the secondary antibody used in the experiment were purchased from Beyotime Technology (China). ML385 was obtained from Selleck (USA). YHR was self-made for this research group.

## Animals

Male Sprague-Dawley (SD) rats (about 260 g in weight) were purchased from Shanghai Slack Laboratory Animal Co., Ltd. (SSCKX 2010 ~ 2011). Rodents are fed with dry feed according to the national standard, and eat freely. The indoor temperature of the animal is 18–22°C, and the relative humidity is 40%-55%. The animal experiment operation was carried out in the ‘Guidelines for the Care and Use of Laboratory Animals’ approved by the Beijing Xiaotangshan Medical Protection Committee.

## HF rat model

The experiment was divided into three groups: sham group, model group and YHR group. The HF rats model was induced by the ligation of the left anterior descending artery (LAD) coronary artery [11]. In brief, the rats were adaptively fed for one week, weighed and performed abdominal anesthesia (5% chloral hydrate, 7 mL/kg), then fixed in the supine position, and left thoracotomy was performed between the second and third intercostal space, approximately from the aortic root. The anterior descending branch of the left coronary artery was threaded and ligated at 1.5 mm to construct HF animal membranes. Rats in the sham operation group only threaded the left anterior descending coronary artery without ligating. The rats in the YHR group received gastric gavage at a daily dose of 1.23 g/kg for 28 consecutive days. Rats in the sham operation group and the model group only received normal saline gavage without other treatments. At the end of the administration, after an overnight fast. After taking the material, fix the heart tissue sample in 4% paraformaldehyde for future experiments.

## Infarct area of heart

The area of myocardial infarction was measured by TTC staining. After 24 hours of reperfusion, rats in each operation group were anesthetized with 100 g/L chloral hydrate, and then quickly opened the chest to expose the heart. First, quickly rinse with 200 ml of normal saline and observe the right atrial appendage until there is a colorless transparent liquid flowing out. Rats were perfused with 250 mL 40 g/L paraformaldehyde phosphate buffer and fixed. Three consecutive coronal sections with a thickness of 2 mm were made, placed in 2% TTC solution for 20 minutes, and then fixed in 4% paraformaldehyde for 10 minutes. The infarct area was analyzed with Image-J software. The formula of infarct area: (myocardial infarction area/left ventricular cross-sectional area) × 100%.

## UTP nick end labeling for terminal deoxynucleotide transfer (TUNEL)

In order to measure the apoptotic activity of cardiomyocytes, the TdT-mediated dUTP notch end labeling method (HJNEL method) was used to detect the in situ apoptosis level of rat heart. Incubate heart tissue sections with TUNEL reagent for 1 hour at room temperature. Then incubate with α-actin antibody (1:750, Abcam) at 4°C overnight. In addition, the nucleus was stained with DAPI. Use a fluorescence microscope (Zeiss) to observe cells that are TUNEL-marked positive in the field of view. And randomly select three non-overlapping fields of view on each slice to take pictures

## Establishment of an oxygen glucose deprivation/recovery (OGD/R) cell injury model

The H9C2 cells were cultured normally to 80% healing. The cells were divided into three groups: control group, OGD/R induction group and YHR group. A sugar-free medium solution was used to simulate glucose deprivation. In the three-gas (94% N_2_; 5% CO_2_; 1% O_2_) incubator, the cell hypoxia environment is simulated. After 6 hours, DMEM with 10% FBS was added to restore oxygen glucose and the culture was continued for 10 hours. Except for the medium containing YHR (300, 500, 700 μg/ml), YHR group added YHR on the basis of model group. The control group was replaced with fresh nutrient solution at 6 and 10 hours.

## MTT to detect cell viability

Use a cell culture plate to culture H9C2 cells normally to 80% healing to induce OGD/R model. The cells were seeded into a 96-well plate at a density of 8 × 10 ^4^ cells per well. Use YHR to process OGD/R model cells. Check the cell viability according to the instructions of the MTT kit. Remove the cell supernatant, add 50 μL 1 × MTT to each well, and incubate at 37°C for 4 hours to reduce MTT to formazan. After 4 hours, aspirate the supernatant, add 150 μL of DMSO solution to each well, and shake for 5 minutes with a plate shaker. Then, a microplate reader (Thermo, USA) detects the OD value of each well at a wavelength of 550 nm.

## DCFH-DA to detect ROS levels

Take well-grown H9C2 cardiomyocytes. When the cell confluence reaches 80% or more, inoculate H9C2 cells in a 6-well plate at a density of 8 × 10^3^ cells/ml, group them according to the experimental requirements, and measure the cell production of ROS. After incubation in different treatments, the H9C2 cells were washed 3 times with PBS, centrifuged, and the pelleted cells were incubated with DCFH-DA (10 μM) serum-free medium at 37°C in the dark for 30 minutes. Then, take pictures under a fluorescence microscope to detect the fluorescence intensity. Image J is used to analyze the data.

## Measuring cell apoptosis with Hoechst 33,342 assay

H9C2 cardiomyocytes are planted in 6-well plates and grow to about 80% for the experiment. The model group was cultured for 8 hours under hypoxia and hypoglycemia, and 12 hours after reoxygenation and hypoglycemia. The cells are divided into three groups: control group, model group, and YHR group. The administration group was treated the same as the model group except that different concentrations of YHR were added during model building. The cells in the normal group were replaced with normal medium at the corresponding time point. Hoechst staining was used to evaluate cell apoptosis after 12 hours of reoxygenation and complex sugar. After different treatments, add the DMEM medium containing Hoechst 33,342 (10 μg/ml) and incubate at 37°C for 15 minutes; discard the supernatant, wash with PBS 3 times, observe the cell apoptosis under a fluorescence microscope, and Take pictures. And count the number of apoptotic cells.

## Fluorescent probe JC-1 to detect mitochondrial membrane potential (ΔΨm)

In the early stage of apoptosis, the mitochondrial membrane potential of the cell will decrease. The fluorescent probe JC-1 can selectively enter the mitochondria, and under the excitation of the excitation light, the fluorescence can change with the change of the mitochondrial membrane potential. The cells are divided into three groups: control group, model group, and YHR group. Add 0.25 ml JC-1 dye solution to each well, shake carefully, and incubate in a 37°C incubator in the dark for 20 minutes. Then wash twice with frozen calcium benchtop buffer. Measure the fluorescence with a flow cytometer.

## Western blotting analysis of Keap1/Nrf2 pathway and apoptosis-related proteins

Through in vivo animal experiments and in vitro cell experiments, the expression of apoptosis-related signal proteins was detected. Extract cell protein according to the operating procedure provided by the reagent manufacturer. The BCA protein determination kit was used to measure the protein content of the solution. Boil with the loading buffer for 5 minutes, and separate the prepared samples with 10% SDS-PAGE separation gel and transfer to NC membrane (Millipore, Germany) under the action of voltage. After washing with PBS, it was blocked in 5% skimmed milk powder, and the protein-laden NC membrane was incubated with different primary antibodies, and placed in a shaker at 4°C overnight. After washing 3 times with TBST, the NC membrane bound to the primary antibody was incubated with the secondary antibody bound to the HRP for 1 hour at room temperature. Wash 3 times with TBST, detect protein with enhanced chemiluminescence agent, and take pictures with image analyzer.

## Statistical analysis

SPSS 17.0 statistical software was used to analyze the data, and the data were expressed as mean ± standard deviation (SD). The comparisons between two groups were performed by Student’s t test. P < 0.05 was considered statistically significant.

## Results

### The influence of YHR on the area of cardiac infarction

**Figure 1.**
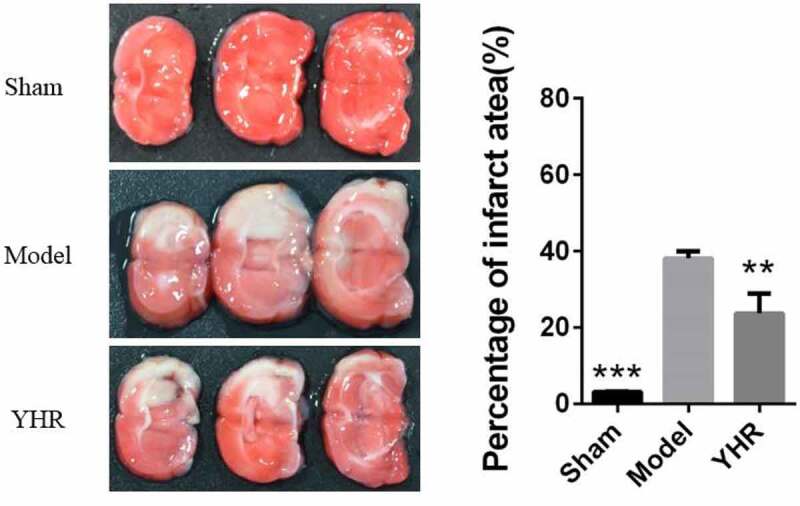
YHR inhibited the area of cardiac infarction. Heart cross section (left) and infarct area (right). Each group consists of three continuous cross sections of rat heart. The ratio of the infarct area to a single section X 100% was used as the infarct rate. The infarction rate is expressed as the mean ± SD of each group. **P  <0.01, ***P < 0.001 vs. Model group

To determine the effect of YHR on the treatment of HF, we used LAD coronary artery to induce an animal model of HF. Twenty-eight days after the operation, the size of myocardial infarction was measured by staining with TTC (Figure 1). In the model group, the heart tissue sections showed a higher infarct size. The sham operation group had very few heart infarctions (3.27 ± 0.12%), and the model group had an increase in the area of heart infarctions (38.13 ± 1.85%). Compared with the HF model group, YHR could reduce the area of heart infarction (23.80 ± 5.06%).

## The effect of YHR on apoptosis and related proteins in HF model

**Figure 2.**
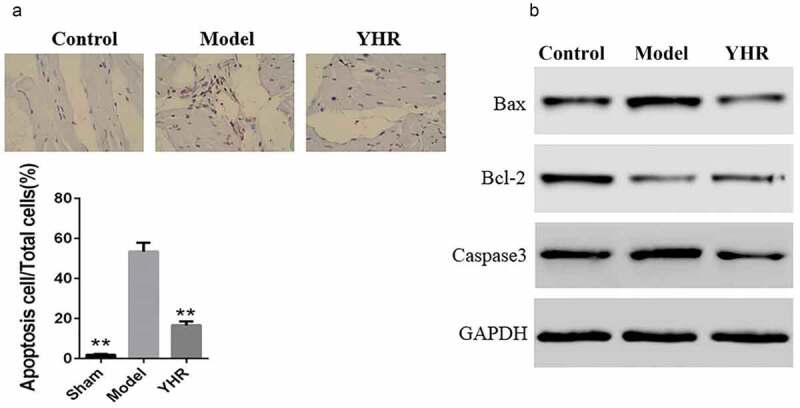
YHR suppressed cell apoptosis of cardiac tissue in HF model

In order to test whether YHR protects heart function by inhibiting apoptosis, we performed TUNEL assay and western blot. The TUNEL assay of cardiac tissue showed that the number of apoptotic cells was higher in the model group, while YHR treatment reduced the number of apoptotic cells (Figure 2a). In addition, the expression of key molecules Bax, Bcl2 and caspase-3 in the apoptosis signal transduction pathway was measured by Western blot. The results showed that, compared with the control group, the expression of Bax and caspase-3 in the HF model group increased, while the expression of Bcl-2 decreased, indicating that heart failure caused the activation of the apoptosis signaling pathway. After YHR was used to treat the HF model group, the protein expression of Bax and caspase-3 was downregulated, and the expression of Bcl-2 was upregulated. These results indicated that YHR could reduce cardiomyocyte apoptosis (Figure 2b).

A. Tunel assay was used to detect the apoptosis rate of the infarct marginal zone of the model group (Magnification, 200×). B. The expression of apoptosis-related proteins, including Bcl-2, Bax and caspase-3 in the cardiac tissue was measured by western blot. **P < 0.01 vs. Model group.

## YHR reverses OGD/R-induced apoptosis

**Figure 3.**
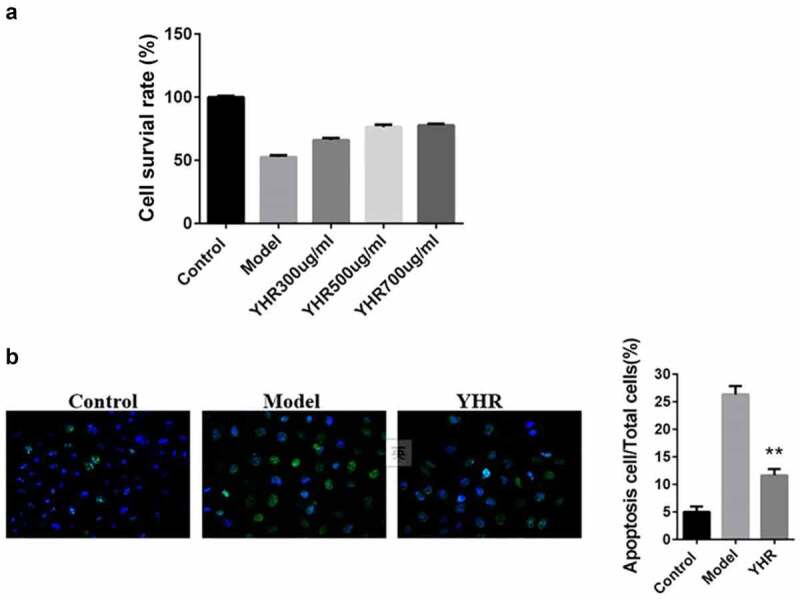
YHR rescued the loss of cell viability induced by OGD/R

To determine the protective effect of YHP on cardiomyocytes in vitro, we used OGD/R to induce H9C2 cell apoptosis model. The MTT and Hoechst 33,342 assay was used to detect the cell viability and apoptosis. Figure 3a shows that OGD/R induction could significantly inhibit the activity of cardiomyocytes. However, YHP (300, 500, 700 μg/ml) obviously promoted the proliferation of cardiomyocytes, and the higher the concentration of YHP, the higher the cell survival rate. Therefore, in subsequent experiments, 500 μg/ml YHR was used to treat cells to evaluate the effect of YHR.

A. H9C2 cells were incubated with different concentrations of YHR (300, 500, 700 μg/ml). MTT assay was used to detect cell viability. B. The cell apoptosis rate was determined by Hoechst 33342 assay (200×). **P < 0.01 vs. Model group.

Figure 3b shows that obvious morphological nuclear changes were observed in the model group under a fluorescence microscope, such as shrinkage of chromatin, production of green fluorescence, condensation and fragmentation of chromatin. The apoptosis rate of the group was significantly higher than that of the control group. YHR treatment can significantly down-regulate the rate of cell apoptosis, suggesting that it has anti-apoptotic properties.

## YHR reduced the loss of mitochondrial membrane potential in cardiomyocytes induced by OGD/R

**Figure 4.**
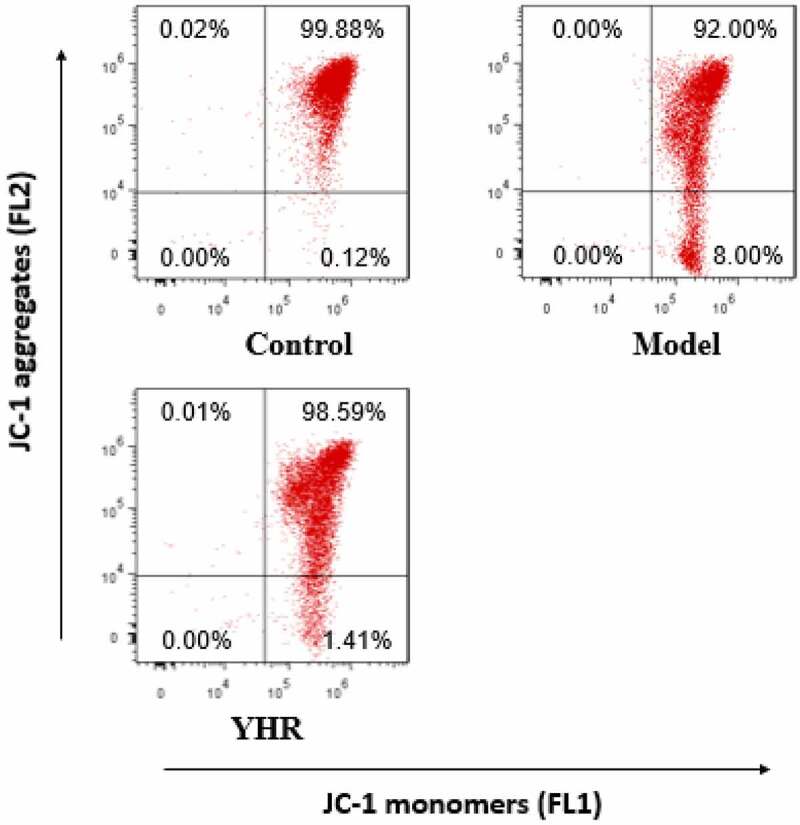
Detect the changes of YHR on the level of mitochondrial membrane potential (ΔΨm) of H9C2 cells induced by OGD/R

In the early stage of apoptosis, the mitochondrial membrane potential of the cell will decrease. Compared with the control group, the mitochondrial membrane potential of H9C2 cells decreased after treatment with OGD/R. After treating with YHR, the loss of cell mitochondrial membrane potential was reduced (Figure 4).

After OGD/R treatment of H9C2 cells, YHR was added and incubated with flow cytometry to detect JC-1 fluorescence intensity.

## YHR attenuates the production of ROS in cardiomyocytes induced by OGD/R

**Figure 5.**
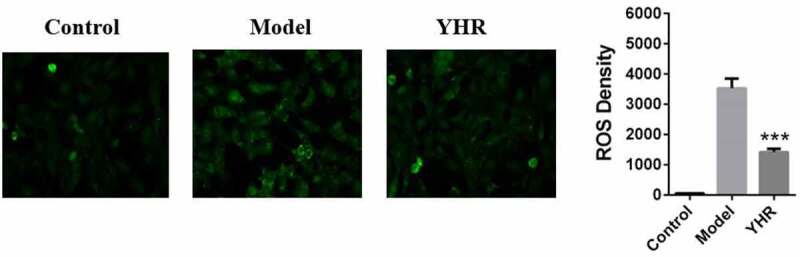
YHR attenuates the production of ROS in cardiomyocytes induced by OGD/R

OGD/R-induced ROS production can promote H9C2 cell damage. On the basis of constructing H9C2 cell damage induced by OGD/R, YHR treatment was added to verify whether YHR can reduce the oxidative stress induced by OGD/R. The level of cellular oxidative stress was measured by the DCFH-DA assay. As shown in Figure 5, compared with the control group, the intracellular ROS of H9C2 cells treated with OGD/R increased significantly. Compared with the cells treated with OGD/R, after YHR treatment, the intracellular ROS was significantly reduced.

The DCFH-DA assay was used to detect the ROS levels of cells in different groups. During the OGD/R process, YHR down-regulated the production of ROS (100×) compared with the model. ***P < 0.001 vs. model group.

## YHR suppressed the activation of Keap1/Nrf2/HIF-1α pathway

**Figure 6.**
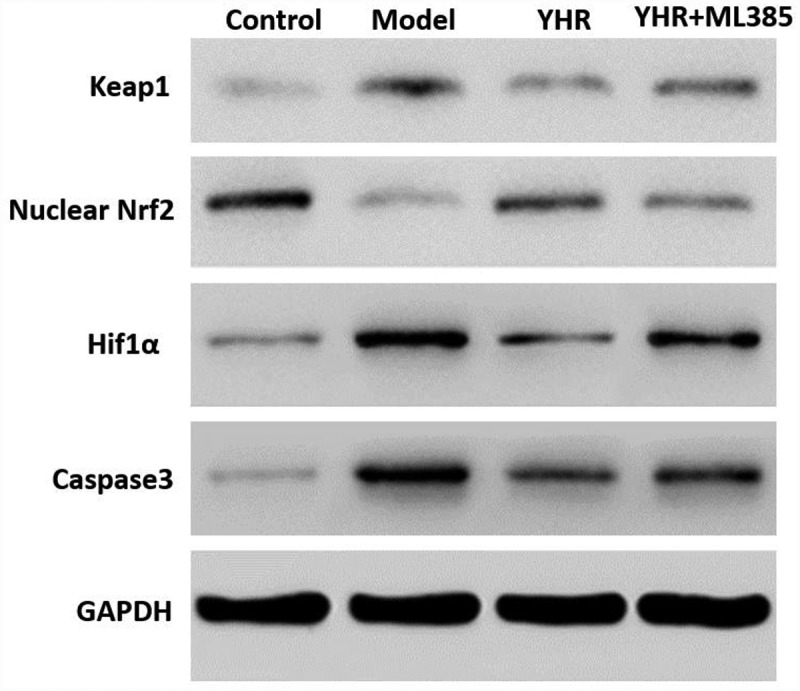
YHR suppressed the activation of Keap1/Nrf2/HIF-1α pathway

Through in vitro cell experiments, the effect of YHR on Keap1/Nrf2 signaling pathway was studied. Western blotting detects the expression of Keap1/Nrf2/HIF-1α pathway-related proteins (Figure 6). Compared with the control group, the expression of nuclear Nrf2 was decreased in the model group, while the expression of Keap1/hif1α/caspase3 was increased. YHR can up-regulate the expression of nuclear Nrf2 and down-regulate the expression of Keap1/HIF-1α/caspase3. However, ML385 abolished the modulating effect of YHR. These results indicate that YHR inhibited apoptosis of cardiomyocytes induced by OGD/R by acting on the Keap1/Nrf2/HIF-1α signaling pathway.

Western blot was used to detect the expression of nuclear Nrf2, Keap1, HIF-1α, and caspase 3. YHR up-regulates nuclear Nrf2 and down-regulates the expression of Keap1/hif1α/caspase3. The co-incubation of H9C2 cells with Keap1-Nrf2 inhibitor ML385 eliminated the regulatory effect of YHR. GAPDH serves as an internal reference protein.

## Discussion

Studies have shown that the loss of cardiomyocytes caused by cardiomyocyte apoptosis is a key factor in the aggravation of heart failure. And then inhibition of ventricular remodeling caused by cardiomyocyte apoptosis can improve the prognosis of heart failure and provide an effective and brand-new way for the prevention and treatment of heart failure [14].

Studies have reported that traditional Chinese medicine can improve cardiac remodeling and cardiac function in patients with chronic heart failure, and has good safety [15]. YHR was reported to have potential anti-apoptotic effects, and a protective effect on HF [11]. In this study, we established an HF model of H9C2 cell induced by OGD/R in vitro and rats induced by LAD coronary artery. Through TTC experiments, we found that YHR inhibited cardiomyocyte apoptosis in the border area of ischemic heart tissue in HF rat model. We further studied the mechanism of YHR through in vitro. YHR could increase the cell viability, inhibit the apoptosis rate and the production of intracellular ROS, up-regulate the mitochondrial membrane potential in H9C2 cells stimulated by OGD/R. ROS is the main substance that induces oxidative stress in the body. Its overproduction can impair cardiac function and increase the susceptibility to arrhythmia. It can also cause cardiomyocyte necrosis and apoptosis through direct toxicity, and it can also trigger an inflammatory response [16]. Apoptosis signals act on mitochondria to cause the mitochondrial membrane potential (MMP, ΔΨm) to decrease or collapse, and then cytochrome C is released into the cytoplasm, which then activates the downstream Caspase cascade [17]. Therefore, our results proved that YHR can protect H9C2 cells from apoptosis induced by OGD/R by inhibiting the generation of ROS and up-regulating the mitochondrial membrane potential.

The Keap1-Nrf2 signaling pathway is one of the cell signaling mechanisms that regulate physiological and pathological processes in animals. It is a popular regulatory pathway of oxidative stress in the body in recent years. Recent studies have shown that Keap1-Nrf2 signaling pathway is effective in resisting oxidative stress damage and anti-inflammatory. It plays an important role in response and anti-tumor [18,19]. Current studies have shown that activating the expression of Nrf2 and its downstream genes can significantly reduce oxidative stress, inflammation, and myocardial cell apoptosis, and significantly alleviate myocardial remodeling caused by hypertension, diabetes, and ischemic cardiomyopathy [20]. In mammalian cells, the ratio of anti-apoptotic proteins (Bcl-2) and pro-apoptotic (Bax) proteins in the Bcl-2 family is critical to cell death. The location of Bax and Bcl-2 in mitochondria is very close, and they can initiate downstream signaling pathways by affecting the permeability of mitochondrial membranes to cause irreversible damage to cells [21]. In this study, the increased expression of caspase-3 indicated that the apoptotic pathway in the H9C2 cell apoptosis model induced by OGD/R and the HF rat model was activated. Western blotting experiments showed that YHR could increase the expression level of Bcl-2, inhibit the expression level of Bax and reduce the expression level of caspase-3. In addition, we also confirmed that YHR inhibited the expression of Keap1 gene. In order to further determine that the mechanism of YHR on apoptosis is through the Keap1/Nrf2 pathway, we selected the Nrf2 inhibitor ML385 to study the effect of YHR on the Keap1/Nrf2/HIF-1α signaling pathway. The Keap1/Nrf2 pathway plays an important regulatory role in cell proliferation and apoptosis. In the last part of the experiment, after adding ML385, the protective effect of YHR was reversed. YHR up-regulates nuclear Nrf2 and down-regulates the expression of keap1/hif1α/caspase3. The co-incubation of cells with ML385 eliminated the regulatory effect of YHR, indicating that ML385 blocked the anti-apoptotic effect of YHR.

## Conclusion

In summary, YHR can prevent cardiomyocyte apoptosis by regulating the Keap1/Nrf2/HIF-1α signaling pathway.

## Article Highlights

1. Yiqi Huoxue Recipe (YHR) reduce infarct area of heart tissue and cardiomyocyte apoptosis rate in HF rats model.
2. YHR inhibit apoptosis of H9C2 cells induced by OGD/R.

YHR suppresses the activation of the Keap1/Nrf2/HIF-1α apoptosis pathway in H9C2 cells induced by OGD/R.

## References

[1] S A H, W T A, M H C, et al. ACC/AHA 2005 guideline update for the diagnosis and management of chronic heart failure in the adult: a report of the American college of cardiology/American heart association task force on practice guidelines (writing committee to update the 2001 guidelines for the evaluation and management of heart failure): developed in collaboration with the American college of chest physicians and the international society for heart and lung transplantation: endorsed by the heart rhythm society. Circulation. 2005;112(12):e154–235. 16160202 10.1161/CIRCULATIONAHA.105.167586

[2] Agustí A, Bonet S, J M A, et al. Adverse effects of ACE inhibitors in patients with chronic heart failure and/or ventricular dysfunction: meta-analysis of randomised clinical trials. Drug Saf. 2003;26(4):895–908. 12959631 10.2165/00002018-200326120-00004

[3] Deng H, Ouyang W, Zhang L, et al. LncRNA GASL1 is downregulated in chronic heart failure and regulates cardiomyocyte apoptosis. Cell Mol Biol Lett. 2019;24(1):41. 31223316 10.1186/s11658-019-0165-x PMC6567419

[4] Y L N, S W A, G T D, et al. Treatment optimization of beta-blockers in chronic heart failure therapy. Sci Rep. 2020;10(1):15903. 32981932 10.1038/s41598-020-72836-4 PMC7522285

[5] Toledo C, D C A, H S D, et al. Neurocognitive disorders in heart failure: novel pathophysiological mechanisms underpinning memory loss and learning impairment. Mol Neurobiol. 2019;56(12):8035–8051. 31165973 10.1007/s12035-019-01655-0

[6] Konishi M, Baumgarten A, Ishida J, et al. Protein levels in Keap1-Nrf2 system in human failing heart. Int J Cardiol. 2016;225:62–64. 27710805 10.1016/j.ijcard.2016.09.128

[7] S H Y, N W Z, Z R G, et al. Modulations of Keap1-Nrf2 signaling axis by TIIA ameliorated the oxidative stress-induced myocardial apoptosis. Free Radic Biol Med. 2018;115:191–201. 29221988 10.1016/j.freeradbiomed.2017.12.001

[8] Ramadori P, Drescher H, Erschfeld S, et al. Hepatocyte-specific Keap1 deletion reduces liver steatosis but not inflammation during non-alcoholic steatohepatitis development. Free Radic Biol Med. 2016;91:114–126. 26698665 10.1016/j.freeradbiomed.2015.12.014

[9] Antognelli C, Trapani E, Delle Monache S, et al. KRIT1 loss-of-function induces a chronic Nrf2-mediated adaptive homeostasis that sensitizes cells to oxidative stress: implication for cerebral cavernous malformation disease. Free Radic Biol Med. 2018;115:202–218. 29170092 10.1016/j.freeradbiomed.2017.11.014 PMC5806631

[10] M C L, Zhao J, Y T L, et al. CPUY192018, a potent inhibitor of the Keap1-Nrf2 protein-protein interaction, alleviates renal inflammation in mice by restricting oxidative stress and NF-κB activation. Redox Biol. 2019;26:101266. 31279986 10.1016/j.redox.2019.101266 PMC6614503

[11] L X L, A M W, D M Z, et al. Yiqi huoxue recipe improves heart function through inhibiting apoptosis related to endoplasmic reticulum stress in myocardial infarction model of rats. Evid Based Complement Alternat Med. 2014;2014:745919. 24864159 10.1155/2014/745919 PMC4016842

[12] Li W-C, Zhao S-X, Ren W-G, et al. Yiqi huoxue recipe improves liver regeneration in rats after partial hepatectomy via JNK pathway. Evid Based Complement Alternat Med. 2020;2020:9085801. 32419833 10.1155/2020/9085801 PMC7201470

[13] C Q T, Di Y, Z Q L, et al. Effect of yiqi huoxue recipe on cardiac function and ultrastructure in regression of pressure overload-induced myocardial hypertrophy in rats. Chin J Integr Med. 2007;13(4):291–296. 18180895 10.1007/s11655-007-0291-6

[14] Takemura G, Kanamori H, Okada H, et al. Anti-apoptosis in nonmyocytes and pro-autophagy in cardiomyocytes: two strategies against postinfarction heart failure through regulation of cell death/degeneration. Heart Fail Rev. 2018;23:759–772. 29737434 10.1007/s10741-018-9708-x

[15] Hao P, Jiang F, Cheng J, et al. Traditional Chinese medicine for cardiovascular disease: evidence and potential mechanisms. J Am Coll Cardiol. 2017;69(24):2952–2966. 28619197 10.1016/j.jacc.2017.04.041

[16] Hou L, Guo J, Xu F, et al. Cardiomyocyte dimethylarginine dimethylaminohydrolase1 attenuates left-ventricular remodeling after acute myocardial infarction: involvement in oxidative stress and apoptosis. Basic Res Cardiol. 2018;113(4):28. 29892894 10.1007/s00395-018-0685-y

[17] H A K, Bazylianska V, M A R, et al. Tissue-specific regulation of cytochromec by post-translational modifications: respiration, the mitochondrial membrane potential, ROS, and apoptosis. Faseb J. 2019;33(2):1540–1553. 30222078 10.1096/fj.201801417R PMC6338631

[18] Cheng Q, Jiang S, Huang L, et al. Zearalenone induced oxidative stress in the jejunum in postweaning gilts through modulation of the Keap1-Nrf2 signaling pathway and relevant genes1. J Anim Sci. 2019;97(4):1722–1733. 30753491 10.1093/jas/skz051 PMC6447257

[19] Li B, Jiang T, Liu H, et al. Andrographolide protects chondrocytes from oxidative stress injury by activation of the Keap1-Nrf2-Are signaling pathway. J Cell Physiol. 2018;234(1):561–571. 30071128 10.1002/jcp.26769

[20] He F, Antonucci L, Yamachika S, et al. NRF2 activates growth factor genes and downstream AKT signaling to induce mouse and human hepatomegaly. J Hepatol. 2020;72(6):1182–1195. 32105670 10.1016/j.jhep.2020.01.023 PMC8054878

[21] K L O, Huang K, Zhang J, et al. Inactivation of prosurvival Bcl-2 proteins activates Bax/Bak through the outer mitochondrial membrane. Genes Dev. 2016;30(8):973–988. 27056669 10.1101/gad.276725.115 PMC4840302

